# Preliminary testing of a patient decision aid for patients with relapsing-remitting multiple sclerosis

**DOI:** 10.1177/20552173211029966

**Published:** 2021-07-15

**Authors:** Nick Bansback*, Judy A Chiu*, Rebecca Metcalfe, Emmanuelle Lapointe, Alice Schabas, Marilyn Lenzen, Anthony Traboulsee, Larry D Lynd*, Robert Carruthers

**Affiliations:** School of Population and Public Health, University of British Columbia, Vancouver, Canada; Centre for Health Evaluation & Outcome Sciences, St. Paul’s Hospital, Vancouver, Canada; Centre for Health Evaluation & Outcome Sciences, St. Paul’s Hospital, Vancouver, Canada; School of Population and Public Health, University of British Columbia, Vancouver, Canada; Centre for Health Evaluation & Outcome Sciences, St. Paul’s Hospital, Vancouver, Canada; Division of Neurology, University of British Columbia, Vancouver, Canada; Patient Partner; Division of Neurology, University of British Columbia, Vancouver, Canada; Faculty of Pharmaceutical Sciences, University of British Columbia, Vancouver, Canada; Collaboration for Outcomes Research and Evaluation, Vancouver, Canada; Division of Neurology, University of British Columbia, Vancouver, Canada

**Keywords:** Multiple sclerosis, disease-modifying therapies, relapsing-remitting, decision-making, decision aid, treatment

## Abstract

**Background:**

Multiple first-line disease modifying therapies (DMTs) are available for relapsing-remitting multiple sclerosis (RRMS), each with different characteristics. We developed an interactive patient decision aid (PtDA) to promote informed shared decision-making (SDM).

**Objective:**

To test the preliminary effectiveness of the PtDA in participants with RRMS.

**Methods:**

Knowledge, and decisional conflict were measured pre- and post- implementation of the PtDA, SDM after the consultation, and 6-month treatment patterns were observed. Differences in scores were analyzed using descriptive statistics and paired t-tests. Qualitative interviews with patients and neurologists were analyzed using thematic analysis.

**Results:**

52 participants were recruited: most were female (81%), 40 years of age or younger (62%), and had experienced MS for less than 5 years (56%). After participants used the PtDA, there was a significant improvement in decisional conflict (change = 1.00; *p* < 0.001) and knowledge (change = 2.15, p < 0.001). Nearly all patients wanted SDM, and 25 (56%) reported this occurred in their consult. Qualitative results suggested the PtDA supported both patients and neurologists in making decisions.

**Conclusion:**

This pilot study suggests that PtDA use helps RRMS patients and their clinician select a DMT. Future studies will assess the feasibility of implementation and the impact of the PtDA on timely DMT initiation and longer-term adherence.

## Introduction

Multiple sclerosis (MS) is a leading cause of non-traumatic neurological disability for young adults.^1–5^ Early initiation of disease modifying therapies (DMTs) with close monitoring is recommended for patients with relapsing-remitting MS (RRMS).^6^ Delaying treatment can cause both increased morbidity and healthcare costs for society.^7^ Numerous different DMTs exist, each with varying administration, effectiveness, side-effect profile, safety and price.^8,9^ In MS, contributors to DMT adherence such as needle phobia and dosing schedule are factors that relate to patient lifestyle and preferences.^10–12^ Adherence to MS DMT is a multifaceted issue^13^ that can impact clinical outcomes.^14^

Patient decision aids (PtDAs) are evidence-based tools that facilitate patient-physician communication,^15^ increase patient knowledge and reduce decisional conflict.^16^ In other medical conditions, PtDAs have been found to increase both treatment initiation and adherence.^16^ Hypothetically, a PtDA could have a similar impact in RRMS^17^ since physicians can be poor predictors of patient priorities.^18–20^

Using the RRMS-PtDA we have previously developed^21^ which meets all 7 out of 7 IPDAS criteria to be defined as a PtDA (Table 1), we sought to assess whether it: 1) reduces patients’ decisional conflict; 2) improves knowledge about MS and DMTs; and 3) improves shared decision-making for patients considering first-line treatment. We also sought to understand patient and physician experiences using the tool as part of routine clinical care.

**Table 1.** IPDAS criteria to be defined as a patient decision aid.

Criteria	Answer
• The decision aid describes the condition (health or other) related to the decision.	Yes
• The decision aid describes the decision that needs to be considered (the index decision).	Yes
• The decision aid identifies the target audience.	Yes
• The decision aid lists the options (health care or other).	Yes
• The decision aid has information about the positive features of the options (e.g. benefits, advantages).	Yes
• The decision aid has information about negative features of the options (e.g. harms, side effects, disadvantages).	Yes
• The decision aid helps patients clarify their values for outcomes of options by: a) asking people to think about which positive and negative features of the options matter most to them AND/OR b) describing each option to help patients imagine the physical, social, and/or psychological effect.	Yes

## Methods

### Sample

Patients were recruited by four neurologists at the University of British Columbia (UBC) Hospital’s MS Clinic in Vancouver, Canada between November 2017 to October 2018. The last participant completed the study in January 2020. Patients were eligible if they: 1) had RRMS; 2) were treatment naïve, considering switching from one to another first-line therapy, or were untreated for two years; 3) could read and speak English; and 4) had internet access. Patients were ineligible if they had a diagnosis of clinically isolated syndrome, primary-progressive MS, or secondary-progressive MS. This study was approved by the UBC Behavioural Research Ethics Board (H16-02302).

### Study design

**Figure 1.**
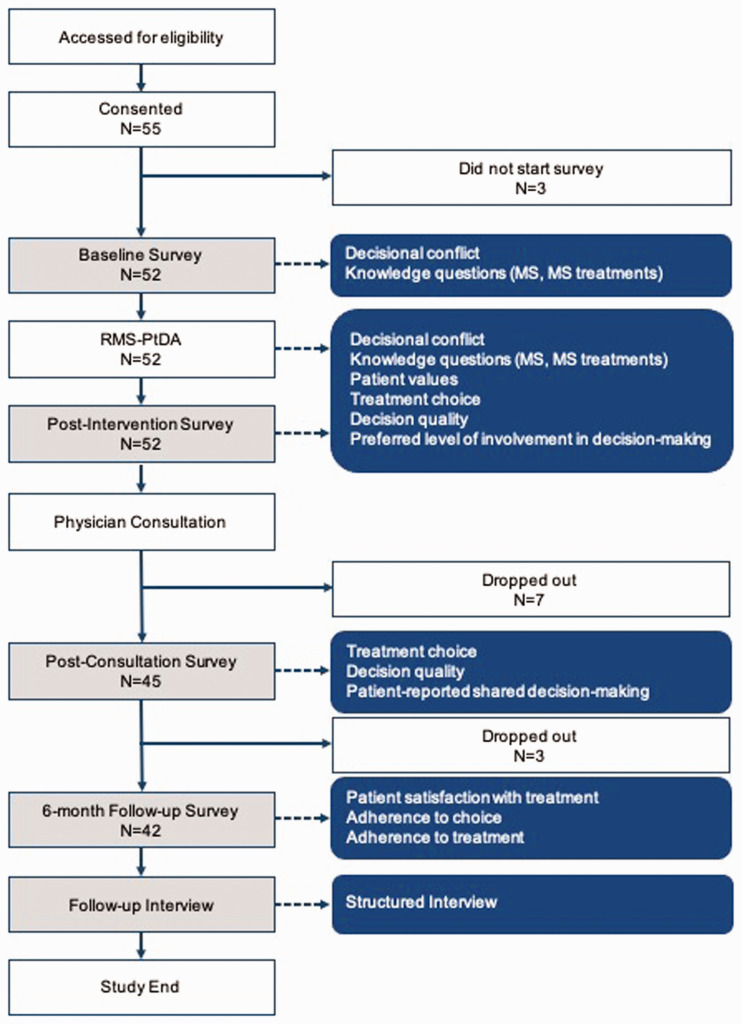
Study design and outcomes collected.

This was a prospective proof-of-concept pre-post study (Figure 1). Patients who met eligibility criteria were provided with a copy of the consent form. Interested participants spoke with a study co-ordinator and provided a signed informed consent form or verbal consent over the phone.

Data were collected at four time points. Participants were sent an email containing a unique URL to the baseline survey (time 1) which collected baseline measures before being directed to the RRMS-PtDA (intervention). Shortly after completing the RRMS-PtDA, participants completed the post-intervention survey (time 2). The RRMS-PtDA produces a one-page summary report which is designed to help patients discuss their questions, concerns, and preferences with their doctor. The summary report was provided to the treating neurologist before each participant’s treatment consultation. After the consultation, participants were sent a unique URL to the post-consultation survey (time 3) to collect information on the treatment decision and the discussion that occurred during the consultation, including extent and quality of shared decision-making. Six months after the treatment consultation, participants were sent a URL to the follow-up survey (time 4) and completed a short telephone interview about their experience using the RRMS-PtDA.

### Patient decision aid (PtDA)

The RRMS-PtDA had five sections (Table 2), which aimed to elicit the patient’s preferences, values, and allowed patients to ask questions they would like to discuss with their neurologist at their next appointment. It is unique in that it individualizes the treatment selection and information in line with the treatment aspects that matter most to the patients.

**Table 2.** Sections of the RRMS-PtDA.

Section	Description
1. History module	To collect information on the patient’s medical history, used to provide personalized information on the following pages (e.g. Patient Determined Disease Steps, an ordinal patient reported outcome of MS patients’ perceived disability and walking ability [0 = normal, 3 = gait disability, 6 = bilateral support, 8 = bedridden]^22^
2. Information module	To present the effectiveness and side-effects of the first-line DMTs and non-medicinal strategies (wellness and lifestyle) to help manage MS
3. Interactive value elicitation module	To guide patients in considering the six most important aspects of treatments for people with RRMS,^23^ which includes:• Slowing progression of MS• Reducing symptoms associated with MS• Preventing relapse and MRI changes• Minimizing minor side effects• Avoiding serious adverse events• Route of administration
4. Decision module	Suggests a treatment that best fits the patient using information from the previous three sections
5. Tailored summary	Summarizes the patient’s health status, preferred treatment choices, and questions they have for further discussion at their following consultation.While the physician was provided with a 1-pag summary of the study procedures and the name and contact information of the research coordinator for assistance, use of the summary page during the consultations were left to the physician’s discretion and clinical expertise.

### Baseline (time 1) measures and post-intervention outcomes (time 2)

The baseline survey (time 1) collected baseline demographics and MS history, and assessed participants’ decisional conflict, knowledge, and preferences for involvement in shared decision-making. The impact of the PtDA was measured by assessing decisional conflict and knowledge again after using the tool (time 2).

#### Decisional conflict

The primary outcome was decisional conflict as measured by the SURE test, a 4-item (yes/no) version of the decisional conflict scale (DCS) for clinical practice.^24^ The DCS measures personal perceptions uncertainty in choosing options, factors contributing to uncertainty, and effective decision-making.^25^ It is internally consistent and reliable (test-retest exceeds 0.78), correlates with the constructs of knowledge, regret and discontinuance, and is able to discriminate between those likely to make a treatment and those who delay treatment decisions.^25^ Decisional conflict may also function as a proxy for longer term treatment adherence.^26–28^ The SURE test score ranges from 0 (extremely high decisional conflict) to 4 (no decisional conflict).

#### Knowledge

Knowledge of MS and DMTs was measured using a German questionnaire^29^ adapted for the Canadian context. The German questionnaire included 19 questions and was found to have good reliability. The adaptation included 7 questions, in which new treatments were added to the responses and questions unrelated to RRMS (e.g. therapies for secondary-progressive MS or double-blind placebo-controlled studies) were removed.

#### Preferences for involvement in shared decision-making

Preference for involvement in decision-making was assessed using the Control Preferences Scale (CPS) which assesses “the degree of control an individual wants to assume when decisions are being made about medical treatment”.^30^ The scale is valid and reliable measure of preferred roles in healthcare decision-making in a variety of populations,^30^ including MS.^31^

### Post-consultation outcomes (time 3)

Treatment choice was determined by one question: “Which treatment option did you and your doctor decide is best for you?” Participants were also invited to respond to two optional open-ended questions about how they decided on the treatment during the consultation with their neurologist, and whether it was the same or different to what was selected on the PtDA and why.

Patient-reported shared decision-making was measured using the Shared Decision-Making Process (SDMP) scale.^32^ The SDMP is a validated tool that measures the extent to which shared decision-making occurred during a patient-provider interaction. It has demonstrated reliability (internal consistency and short-term test-retest reliability) and strong construct validity. It is comprised of 4 questions with 2 (yes/no) or 4 (a lot/some/a little/not at all) response categories. Scores range from 0 to 4 points, where a higher score indicates more involvement in the decision. Shared decision-making was rated as having occurred during the consultation if a participant endorsed 3 or more items.

### Six-month outcomes (time 4)

Six months after the consultation (time 4), participants completed the follow-up survey and reported which treatment they were using. The outcome adherence to choice^33^ was defined as the proportion of participants who adhered to the choice they made with their neurologist during their treatment consultation. Participants were considered to be adherent if they have not discontinued therapy (defined as participants’ self-report of stopping therapy for >30 days, or if detected before 30 days since discontinuation, self-reported intention to permanently stop therapy) and was measured as the proportion of participants adherent.

Satisfaction was assessed using a single question: “How satisfied or dissatisfied are you with this medication?” Responses ranged from extremely dissatisfied to extremely satisfied.

### Qualitative interviews

#### Participants

After the follow-up survey, participants completed a semi-structured telephone interview with the research assistant. Interview questions explored participant experiences using the PtDA broadly and sought specific feedback on: 1) the RRMS-PtDA as an educational tool, 2) factors that contributed to their treatment decision with their neurologist, 3) the use of decision aids in MS care, and 4) how the tool could be improved.

#### Physicians

Three treating physicians also completed a semi-structured interview to share their perceptions on how the RRMS-PtDA helped prepare their patients, how it helped them understand their patients’ concerns, and feedback on how it can be improved for clinical use.

### Analysis

Descriptive statistics were used to assess participant characteristics as well as scores from the SURE test, the knowledge questionnaire and the SDMP. Differences in scores were assessed using paired t-tests.

Qualitative data from the follow-up interviews were transcribed and analyzed using thematic analysis and open coding. Codes were grouped into themes and then reviewed by an independent researcher who did not participate in the surveys or the interviews. Any coding concerns were resolved by discussion.

## Results

### Sample characteristics

Fifty-five participants enrolled in the study; 52 patients (95%) completed the baseline survey, the PtDA, and the post-intervention survey and were included in the analysis. Forty-five patients (87%) completed the post-consultation survey and 43 (83%) completed the 6-month follow-up survey (Figure 1). At baseline, the majority of participants were female (81%), less than 40 years of age (62%), had mild disability (87% had a PDDS of 2 or less) and experienced MS symptoms for less than 5 years (56%) (Table 3). 75% of participants were treatment naïve, and 77% had private insurance.

**Table 3.** Baseline and clinical characteristics (N = 52).

Characteristic	n (%)
Age (years)	
30 or younger	13 (25)
31–40	19 (37)
41–50	14 (27)
51–60	5 (10)
61+	1 (2)
Sex, Female	42 (81)
PDDS	
Mild disability (0-2)	45 (87)
Moderate disability (3–5)	6 (12)
Severe disability (6–8)	1 (2)
Years experiencing MS symptoms	
0 to less than 2 years	14 (27)
2 to less than 5 years	15 (29)
5 to less than 10 years	5 (10)
10 or more years	18 (35)
Reported at least 1 relapse in the last 2 years	41 (79)
Reported an MRI with new MS lesions in the last year	36 (69)
Treatment naïve	39 (75)
Has private insurance on top of B.C. Pharmacare	40 (77)
Control preferences scale	
I prefer to make the decision about which treatment I will receive	1 (2)
I prefer to make the final decision about my treatment after seriously considering my doctor’s opinion	25 (48)
I prefer that my doctor and I share responsibility for deciding which treatment is best for me	21 (40)
I prefer that my doctor makes the final decision about which treatment will be used, but seriously considers my opinion	5 (10)
I prefer to leave all decisions regarding treatment to my doctor	0 (0)
Values	
How effective are DMTs at slowing disease progression?	43 (83)
How effective are DMTs at reducing the frequency/ severity of relapses and new MS lesions?	42 (81)
What rare but serious adverse events do I need to be aware of?	41 (79)
How common are serious adverse events that might cause me to withdraw from therapy?	29 (56)
What are the common minor side effects of DMTs?	20 (38)
How are DMTs administered?	10 (19)
When was the DMT approved by Health Canada?	9 (17)
Side effects patients want to avoid most	
Depression / Mild increase in risk of depressive symptoms	27 (52)
Hair thinning or hair loss (reversible)	19 (37)
Gastrointestinal symptoms	15 (29)
Flushing	9 (17)
Injection site reactions	8 (15)
Flu-like symptoms	6 (12)

PDDS: patient determined disease steps; DMT: disease modifying therapy.

### Baseline (time 1) measures and post-intervention (time 2) outcomes

At baseline, participants had a mean decisional conflict score of 1.69 (95% confidence interval (CI): 1.32, 2.05), with 20% of participants reporting a score of 0 (extremely high decisional conflict) and 16% of participants reporting a score of 4 (no decisional conflict) (Table 4). The mean knowledge score was 3.15 (out of 7, 95% CI: 2.87, 3.44). Questions about DMTs were most likely to be answered incorrectly (e.g., identifying which medications increase risk of developing progressive multifocal leukoencephalopathy).

**Table 4.** Decisional conflict and knowledge at baseline and post-intervention.

Score	Pre-n, yes (%)	Post-n, yes (%)	Difference	p-value
Decisional conflict (N = 51)					
Sure of myself	16 (31)	23 (45)	–	–
Understand information	18 (35)	43 (84)	–	–
Risk-benefit ratio	27 (53)	39 (76)	–	–
Encouragement	25 (49)	35 (69)	–	–
Mean (SD)	1.69 (1.35)	2.69 (1.26)	1.00 (1.57)	<0.001
Knowledge (N = 52)	n, correct (%)	n, correct (%)		
What are relapses?	51 (98)	51 (98)	–	–
When can a diagnosis of MS be made?	50 (96)	52 (100)	–	–
What is the general effect of disease modifying therapies?	34 (65)	21 (40)	–	–
Which DMTs are administered by self-injections?	17 (33)	28 (54)	–	–
Compared to beta-interferons, what is the effect of Copaxone on relapse rates?	8 (15)	48 (92)	–	–
If 100 patients start an interferon treatment, how many would have flu-like symptoms in the beginning?	2 (4)	51 (98)	–	–
Which DMT(s) put you at an increased risk of developing PML?	2 (4)	25 (48)	–	–
Mean (SD)	3.15 (1.02)	5.31 (1.42)	2.15 (1.58)	<0.001

MS: multiple sclerosis; DMT: disease modifying therapy; PML: progressive multifocal leukoencephalopathy.

Forty-four participants (85%) finished the PtDA on the day it was first accessed. Among these, the majority spent less than one hour using the PtDA (n = 40; mean time 49.1 minutes; IQR 23.3 to 44.7). Four participants spent more than 6 hours using the PtDA.

About 80% of participants indicated that the effectiveness of DMTs in slowing disease progression, reducing the frequency/severity of relapses and new MS lesions, and rare but serious adverse events mattered most to them, being selected more frequently and rated more important. Other attributes were important to a lesser degree, including how common serious adverse events might lead to therapy withdrawal (56%), common minor side effects (38%), route of administration (19%), and when approval from Health Canada was received (17%).

After the PtDA, decisional conflict improved from a mean score of 1.69 to 2.69, a change of 1.00 (p < 0.001), with most patients feeling sure about the benefits and risks of each option (84%) and being clear about which benefits and risks matter most to them (76%). Knowledge scores increased to 5.31 (SD = 1.42), an improvement of 2.15 (p < 0.001) (Table 5). Scores improved for 83% (43/52) of participants, with about half of the participants answering two or more additional questions correctly (25/52).

**Table 5.** Post-consultation outcomes (N = 45).

Patient-reported shared decision-making	N (%)
Did your doctor talk about disease modifying therapies as an option for you?	
Yes	37 (82)
No	8 (18)
How much did you and your doctor talk about the reasons you might want to take a disease modifying therapy?	
A lot	12 (27)
Some	14 (31)
A little	12 (27)
Not at all	7 (16)
How much did you and your doctor talk about the reasons you might not want to take a disease modifying therapy?	
A lot	5 (11)
Some	11 (24)
A little	14 (31)
Not at all	15 (33)
Did your doctor ask you if you wanted to take a disease modifying therapy?	
Yes	29 (64)
No	16 (36)

Almost all (88%) participants preferred a collaborative approach to choosing a treatment, with 48% of participants preferring to make the final decision about treatment after seriously considering their doctors opinion, and 40% preferring to share responsibility for deciding treatment.

### Post-consultation (time 3)

**Figure 2.**
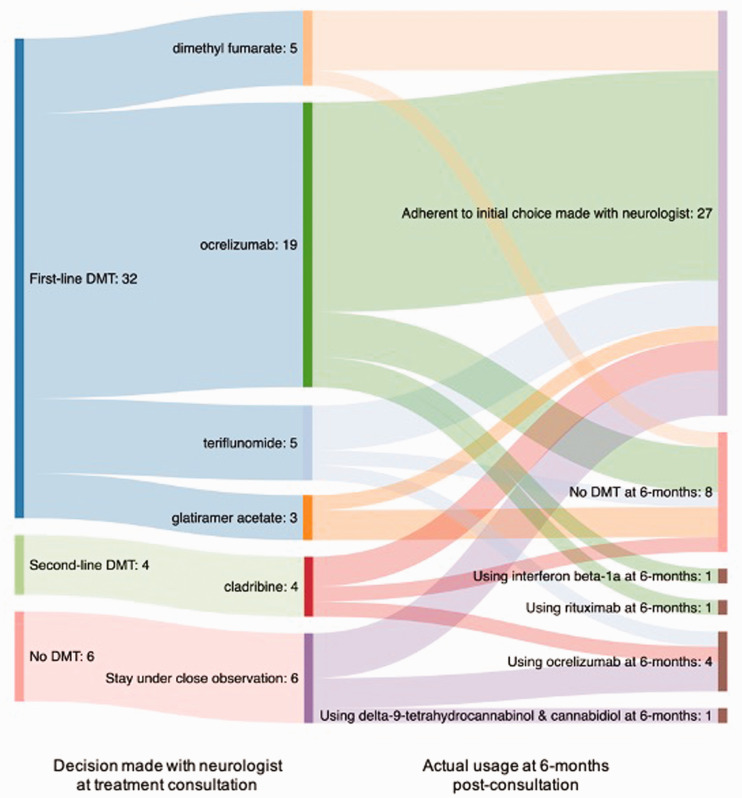
Choice of medication at consultation and after 6-months post-consultation.

Forty-five participants completed the post-consultation survey. After the consultation, 36 (80%) reported choosing a DMT with ocrelizumab (20, 54%) being the most popular (Figure 2). Factors that contributed to the treatment decision included current clinical symptoms, radiographic activity, the doctor’s opinion on the best medication, whether the doctor’s recommendation and the recommendation from the PtDA were the same, whether the patient had insurance coverage, the efficacy of the treatment at preventing further disability and slowing the effects of MS, the side-effect profiles and their tolerability to the patient, weighing the benefits and the risks of each treatment, and whether the medication fit into the patient’s lifestyle.

Twenty-five (56%) participants reported that shared decision-making took place during their consultation with their treating neurologist. Participants reported that DMTs were discussed as an option for them 82% (n = 37) of the time, while only 64% (n = 29) reported being asked by their doctor if they wanted to take a DMT. Reasons the participant might not want to take a DMT were discussed less frequently (35%, n = 16) than the reasons the participant might want to take a DMT (58%, n = 26).

### Six-month follow-up (time 4)

Forty-two participants completed the follow-up survey. Twenty-seven participants (64%) were following the choice that they made with their neurologists at the consultation (Figure 2). Of those that did not, reasons included not qualifying for insurance coverage and using another drug, side-effects leading to discontinuation, or a follow-up MRI indicating worsening lesions. Regardless of whether participants were taking a medication to manage their MS, all but five participants reported making lifestyle changes, including taking vitamin D supplements, changing diet, quitting smoking, increasing exercise and improving stress management.

Of those taking a medication, 25 out of 31 (81%) participants were satisfied, very satisfied, or extremely satisfied with the medication they are taking. Five (16%) were somewhat satisfied and one (3%) was extremely dissatisfied. This was similar to those who decided to stay under close observation and not take medication: 9 out of 12 (75%) participants were satisfied, very satisfied, or extremely satisfied with their current lifestyle changes to manage their MS; while 2 (17%) were somewhat satisfied and 1 (8%) was very dissatisfied.

### Qualitative interviews

#### Participants

Forty-two participants completed interviews 6-months after the treatment consultation. Three themes emerged (see Table 6). The first theme focused on how the PtDA process improved the consultation. Participants shared that the PtDA helped the physician better understand their priorities and needs and helped the participants prepare questions for their physician. In the second theme, participants emphasized the that the PtDA facilitated decision-making by creating structure through its step-by-step process. Participants felt the PtDA made them more informed about the options available and reported that they spent more time considering how each medication may impact their lifestyle than they would have without the PtDA. In the third theme participants reported the emotional benefits of the PtDA. They shared that the decision process was overwhelming but the format and content of the PtDA helped alleviate those feelings and made it easier for them to learn about the options available.

**Table 6.** Qualitative feedback and recommendations from participants.

Theme	Feedback	Recommendations
Improving the consultation	*“So I like that it was hands on and you could decide for yourself, but then I specifically wrote a note in mine that was I want my doctor to tell me. So it kind of provided both sides. It kind of allowed my doctor to understand that I wanted his medical experience and knowledge to be part of my decision.”* *“I really liked that when I made my note [in the decision aid], Dr. X had that when I went to my meeting. He followed that… and he didn’t make a decision for me, but he was what I asked for. It was really validating because he utilized [the decision aid summary] and acted on it.”* *“It’s something [that] gives the doctors and the team know how [the patient is] feeling about the medication. It’s like a briefing, basically, getting to know the patient more. I think it’s a thing they must do.”*	Patients wanted to see the decision aid be part of routine clinical care in the decision-making process; let patients know before their first appointment that this is a tool that is available when the patient is ready to consider treatment options.
Facilitating decision-making	*“It was good because one thing it did do was make me rationalize the choices that I was making, like be clear to myself why I was making the choice that I was making rather than just, I don’t know what so I’ll just pick something, so that was useful.”* *“I found it pretty helpful, how it took you through the process step-by-step, especially because in my experience my doctor didn’t really do that all that efficiently in my opinion, so being able to kind of go through those steps, seeing what the results were, and then being able to compare that to what my doctor and I had talked about was really good. It kind of gave me a little bit more confidence in what I was thinking.”* *“I liked how it showed your first and second choices on the summary sheet. I liked how it created structure. You’re overwhelmed after the first meeting, and with this structure, it helps us consider what treatments you need to consider.”* *“For me, I felt that my direction wasn’t very set. I didn’t feel like I was taken care of at the level of decision-making for my medication that I wanted to be and so, for me, having the option to take something like the patient decision aid is amazing because it’s like okay, great, there’s this resource outside of my doctor that I can use, that can help me in a way that perhaps the doctor isn’t able to or willing to.”* *“It helped me learn more about the benefits and risks and it sort of came up with the same suggestion in the top three – it was the same ones that we were talking about that we sort of researched and came up with and then when we did [the patient decision aid] it kind of confirmed that that was probably the route we wanted to go.”* *“What was most convenient to me and what I was kind of looking for in medication. That was really nice because I actually hadn’t given that much thought before that. I was just going to do whatever was prescribed. I never thought about how it was going to impact my life so that was really quite nice to be able to think about that.”* *“When I was first hearing about potential options, my doctor said would you be willing to stick yourself with a needle every day? And out of complete desperation because potentially those would be safer, well no, but I would if I had to. It was nice to go on the decision aid and look at it and really think about my lifestyle and be like, okay, that doesn't work for me, but here are these other options that I could do, that would fit my lifestyle better.”* *“It’s good to have this decision aid. I liked the extra links [for more information], not emotionally engaging, and that it was online. It’s different to receiving a sheet of paper or a brochure.”*	
Making the decision less overwhelming	*“I wish I had done it initially when I was first diagnosed but I think I was in denial and I didn’t want to accept it so I kind of hesitated and prolonged it. Going back, I wish I had done it right away. That way, I would know what I’m looking at and when someone’s talking to me, like Dr. X telling me about this medication, then I’m a step ahead and I know what he’s talking about.”* *“It’s super overwhelming to have a diagnosis, be told that you need to take medication, and then have so much to choose from. Being able to kind of answer questions and feel like those questions are leading you towards some kind of answer, at least narrowing it down, was nice.”* *“I would love to go through that for all of my treatment drugs that I’m on for my back injuries and stuff because half of the drugs I’m on, I have no idea what they do or anything… what dosages are available so I know less about the drugs I’ve been on for 10 years than the one I’ve taken once.”*	Patients wanted to see more information in the following areas: • What to expect, or not expect, from treatment medication • How medication and lifestyle changes can work together to help manage MS • Why it might be important to go on treatment, but also why some people may not want to go on treatment right away

It is important to note that most participants had one or two medications recommended to them by their neurologist before accessing the PtDA. A small number of participants noted that they did not feel that the PtDA helped them make the decision about using a DMT but helped them feel more confident in the choice that they were leaning towards.

Although most participants found the PtDA easy to use, requested improvements included content in three areas: 1) expectations for treatment medication, 2) how medication and lifestyle changes work together to help manage MS and 3) whether to proceed immediately with medication or defer treatment. Some suggested including a free-text response for certain questions to provide more details. Patients generally suggested the PtDA should be included in routine clinical care.

#### Physician feedback

Three neurologists provided feedback at the end of the study. They felt the RRMS-PtDA helped prepare participants for their visits. For example, one neurologist expressed it was easier to trust patients who chose to defer any treatment since they were more knowledgeable of their options and consequences, and able to articulate specific reasons for not wanting to take DMTs. It also helped neurologists better understand the patient’s priorities.

In terms of improvement, one neurologist suggested providing more specific information for adverse events as *“patients could not really sort out how bad the adverse events really are.”* Participants were, for example, overestimating the risk and impact of infusion reactions related to ocrelizumab. Another neurologist had a challenging time identifying which patients had completed the PtDA and suggested including the decision summary into participants’ EMR as a report that required physician sign-off. This would allow physicians to more easily identify participants who completed the PtDA and serve as a reminder to review the summary report.

## Discussion

In this prospective study we found a PtDA for RRMS facilitated shared decision-making between patients and neurologists around treatment decisions. All but one participant reported wanting shared decision-making highlighting the need for the PtDA. After completing the RRMS-PtDA, participants reported reduced decisional conflict and improved knowledge scores, which suggests that the PtDA helped patients learn about the DMTs and allowed them to feel more confident about their decision. Without a control arm, we cannot evaluate the impact compared to historical care, though the qualitative findings clearly signal that this changed the typical clinical approach.

We were not able to calculate decision quality as an outcome as originally planned, defined if the patient is both informed and chooses a treatment aligned with patient values. With the multitude of DMTs, an evolving disease process in RRMS which can alter which DMTs are appropriate, and differential access to treatments, it was challenging to match values with what were appropriate DMTs for each patient. Instead, this study focused on patient knowledge of DMTs and the quality of patient-physician consultations. According to the clinicians in this effort, patients were more engaged and informed at the point of selecting DMT after completing the PtDA. Participants reported that because they could complete the PtDA on their own time at home, they felt less overwhelmed during the consultation and were better able to ask the questions that were important to them. In a future measure of decision quality, it would be important to consider those who choose not to have treatment (despite demonstrating improved knowledge), as well as other metrics such as anxiety and depression.

Strengths to this study include the PtDA and study design was co-produced with patients and neurologists to ensure it could fit within routine care, and that both patients and neurologists had time with the PtDA and summary report respectively prior to the consultation to prepare. These aspects have been shown to be important for implementation of PtDAs.^34^ The online PtDA allowed participants to rate their preferences and treatment goals interactively sorting the treatments in accordance to what would most likely be preferred – potentially reducing the amount of information that patients would have to read. To our knowledge, this is the first RRMS patient decision aid that has tested the preliminary efficacy. Limitations of this study include the small sample size, single site, lack of a control group, and a rapidly evolving therapeutic landscape in MS. While a randomized controlled trial (RCT) is ideal, we sought to determine characteristics to guide a future RCT and to introduce the PtDA to the physicians as part of routine care using the limited funds available. Ocrelizumab was added in the midst of participant recruitment. However, because this was an online tool, we were able to update the PtDA in a timely manner to reflect availability of drugs emerging from the pipeline. In our cohort, ocrelizumab was a common choice for first-line RRMS, which may reflect the preferences of the treating neurologists. Patient use of ocrelizumab was also dependent on insurance approval. In some cases, drug access changed DMT choice. In others, there were prolonged delays between the initial consultation and the treatment consultation. Evaluations of psychosocial dynamics such as the Hospital Anxiety and Depression Scale could add to future studies.

In conclusion, this single center, prospective evaluation of a PtDA in a RRMS cohort, there was reduced decisional conflict and improved in DMT knowledge. Most participants chose to take a DMT. While the PtDA cannot supplant discussion with the treating neurologist, it may support patients initiating and adhering to a DMT for RRMS. Further study is required, which could include multicenter evaluation of the PtDA in other MS clinics with a control arm.

## Acknowledgements

We thank the participants and neurologists for taking part in this study.

## Conflict of Interests

The author(s) declared the following potential conflicts of interest with respect to the research, authorship, and/or publication of this article: AT reports grants and personal fees from Roche, grants and personal fees from Sanofi Genzyme, personal fees from Biogen, personal fees from Novartis outside the submitted work. EL has received consulting fees from Novartis, Biogen, BMS, Alexion, Genzyme, Hoffman La-Roche and EMD Serono. RC reports grants and personal fees from Roche Canada, grants from Novartis, grants from Novartis, grants from Teva Innovation Canada, grants from EMD Serono, and grants from MedImmune outside the submitted work. NB, JAC, RM, AS, ML, and LL have no disclosures.
